# Multiple Access-Enabled Relaying with Piece-Wise and Forward NOMA: Rate Optimization under Reliability Constraints

**DOI:** 10.3390/s21144783

**Published:** 2021-07-13

**Authors:** Farnaz Khodakhah, Aamir Mahmood, Patrik Österberg, Mikael Gidlund

**Affiliations:** Department of Information Systems and Technology, Mid Sweden University, 851 70 Sundsvall, Sweden; aamir.mahmood@miun.se (A.M.); patrik.osterberg@miun.se (P.Ö.); mikael.gidlund@miun.se (M.G.)

**Keywords:** cooperative communication, NOMA, piece-wise and forward (PF), relaying protocols, rate optimization, QoS

## Abstract

The increasing proliferation of Internet-of-things (IoT) networks in a given space requires exploring various communication solutions (e.g., cooperative relaying, non-orthogonal multiple access, spectrum sharing) jointly to increase the performance of coexisting IoT systems. However, the design complexity of such a system increases, especially under the constraints of performance targets. In this respect, this paper studies multiple-access enabled relaying by a lower-priority secondary system, which cooperatively relays the incoming information to the primary users and simultaneously transmits its own data. We consider that the direct link between the primary transmitter–receiver pair uses orthogonal multiple access in the first phase. In the second phase, a secondary transmitter adopts a relaying strategy to support the direct link while it uses non-orthogonal multiple access (NOMA) to serve the secondary receiver. As a relaying scheme, we propose a piece-wise and forward (PF) relay protocol, which, depending on the absolute value of the received primary signal, acts similar to decode-and-forward (DF) and amplify-and-forward (AF) schemes in high and low signal-to-noise ratio (SNR), respectively. By doing so, PF achieves the best of these two relaying protocols using the adaptive threshold according to the transmitter-relay channel condition. Under PF-NOMA, first, we find the achievable rate region for primary and secondary receivers, and then we formulate an optimization problem to derive the optimal PF-NOMA time and power fraction that maximize the secondary rate subject to reliability constraints on both the primary and the secondary links. Our simulation results and analysis show that the PF-NOMA outperforms DF-NOMA and AF-NOMA-based relaying techniques in terms of achievable rate regions and rate-guaranteed relay locations.

## 1. Introduction

To meet the rapidly increasing demands of Internet-of-things (IoT) services and applications in various domains (e.g., smart building, automation, and city), future wireless networks (i.e., 5G-and-beyond systems) need to address many challenges [1,2]. Notably, the congestion resulting from the proliferation of IoT networks requires a combination of cooperative communication [3], non-orthogonal multiple access (NOMA) [4], and spectrum sharing/coexistence [5] schemes to provide spectral efficient, massive and reliable connectivity. Cooperative communication, or cooperative relaying, exploits the interaction among the network devices in proximity to boost reliability, coverage, and energy efficiency [6,7]. Cooperative relaying schemes provide a spatial diversity, especially when the direct links suffer from the wireless channels’ adverse effects (i.e., path losses, fading, shadowing) or have transmit-power constraints [8,9]. Meanwhile, NOMA has received significant attention for meeting spectrum efficiency, massive connectivity, and low latency demands [10]. Using superposition coding, NOMA combines and transmits signals of multiple users simultaneously over the same time and frequency resource while the users with strong channel conditions employ successive interference cancellation (SIC) to decode and cancel the information of other users with weak channel conditions. By relaying this decoded information to the weak users, cooperative NOMA-based relaying schemes are proposed in the literature to improve reliability and coverage in 5G networks [11]. On the other hand, the spectrum sharing enables multiple networks to coexist in licensed or unlicensed frequency bands with protection guarantees to the primary radio systems [12,13]. By combing these paradigms, the concept of NOMA-aided cooperative cognitive radio (CR) networks is emerging in which a secondary IoT network performs cooperative relaying for the primary system as well as satisfies its connectivity requirements using an overlay mode of spectrum sharing [7]. This approach has been attractive to enable device-to-device (D2D) communications in 5G cellular/heterogeneous networks or reliable coexistence of radio networks in unlicensed bands (e.g., WiFi, Zigbee, Bluetooth) [13,14,15,16,17,18].

Specifically, in [13], a cooperative CR-NOMA scheme is proposed to improve the system performance in terms of outage probability. In [14], the authors focused on optimal relay selection policy in CR-NOMA networks and investigated two different relay selection schemes, namely partial relay selection (PRS) and opportunistic relay selection (ORS). In each scheme, the authors used amplify-and-forward (AF) as a relay protocol to send the incoming superimposed NOMA signal to the destination. Note that, in an AF scheme, the relay node instead of decoding amplifies the received signal before forwarding it. Another challenging problem in cooperative CR-NOMA networks is user scheduling. In this respect, Lu et al. in [15] studied outage probability, diversity, and mutual outage probability of a multicast cognitive radio network (MCR-NOMA) system, using three different user scheduling strategies with decode-and-forward (DF) relaying protocol. In a DF relaying scheme, a relay node first decodes the received signal, then encodes and transmits it forward. They also showed that a cooperative CR-NOMA system’s performance could be improved using a dynamic scheme that switches between cooperative NOMA and cooperative OMA. From an energy-efficiency perspective, the authors in [16] studied the outage performance and the throughput of cooperative CR-NOMA with energy harvesting (EH) under the DF relay strategy. In all these studies mentioned above on cooperative CR-NOMA, (i) either DF or AF protocol is used as the relay protocol, and (ii) rate performance of the system has not been studied.

In cooperative NOMA relaying schemes, choosing a suitable relay protocol is important for system performance; a proper relaying scheme can significantly enhance the system capacity [6] and outage probability [19]. In [6], cooperative CR-NOMA scheme (by using joint OMA and NOMA) is investigated with respect to AF and DF relaying protocols; however, the analysis in [19] is limited to a downlink D2D NOMA system. The authors in [6] showed that AF relaying protocol performs better than the DF protocol in terms of achievable rate regions under optimized allocation of time and power fractions in the joint OMA and NOMA scheme.

Besides choosing an appropriate relaying protocol, the design and optimization of a cooperative relaying scheme with rate guarantees is a challenging task, especially in the primary–secondary coexisting system (i.e., cooperative CR-NOMA system as studied in [6,13,14,15,16,17,18]). In this paper, motivated by the results in [6,19], we study different relaying protocols, including AF, DF, and piece-wise and forward (PF) in such cooperative networks. PF protocol finds its origin in the estimate and forward (EF) protocol, which is shown to be the best relay protocol in the conventional cooperative relaying systems in terms of bit error rate (BER) [20]. However, extracting the probability density function (PDF) of the signal is difficult because of the complicated EF function. To solve this problem, the authors in [21] proposed the PF function as an approximation of EF. In PF relay protocols, the signal transmitted by the relay depends on the absolute value of the incoming signal, while PF acts similar to the DF and AF protocol in high SNR and low SNR, respectively. In this respect, our main contributions in this article are:

- **PF-NOMA**: We propose a PF-NOMA scheme—NOMA-based cooperative relaying scheme using PF protocol—for a coexisting primary–secondary IoT setup, and develop rate formulations for the two systems considering their cooperation time and power allocations within a time slot under the adaptive threshold of PF protocol.
- **Rate optimization under reliability constraints**: We formulate a rate maximization problem for the secondary link under reliability (i.e., guaranteed rate) constraints of both the primary and secondary links. We obtain the optimal time splitting and power splitting factors by decomposing the original optimization problem into two sub-problems leading to closed-form feasible boundary regions and developing an algorithm.
- **Results**: Based on extensive simulations, our analysis shows that, using the PF-NOMA scheme with optimal time-/power-splitting, the secondary link can achieve a higher rate than the baseline approaches (i.e., AF-NOMA and DF-NOMA). Moreover, PF-NOMA provides a higher degree-of-freedom in relay-location selections to support the primary and secondary rate requirements compared to the baseline schemes.

The rest of this paper is organized as follows. Section 2 presents the system model, problem statement, assumptions, and the rate formulation for PF-NOMA. The optimization problem of PF-NOMA is presented in Section 3. The simulation results are discussed in Section 4. Finally, we conclude the article in Section 5.

## 2. System Model

**Figure 1:**
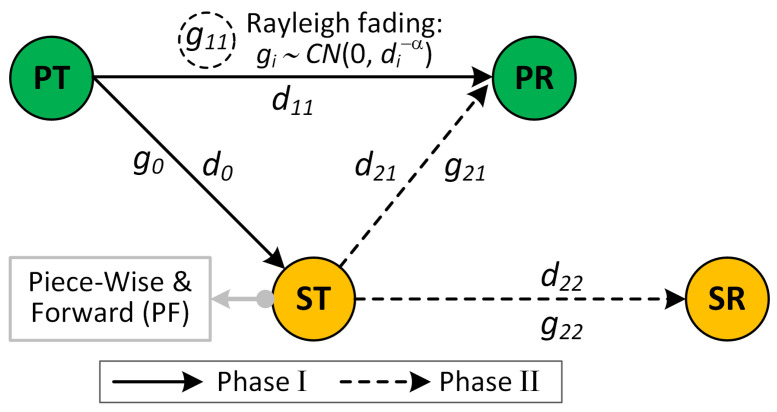
The cooperative network consisted of a primary user and secondary user. The secondary transmitter used PF as a relaying protocol.

**Figure 2:**
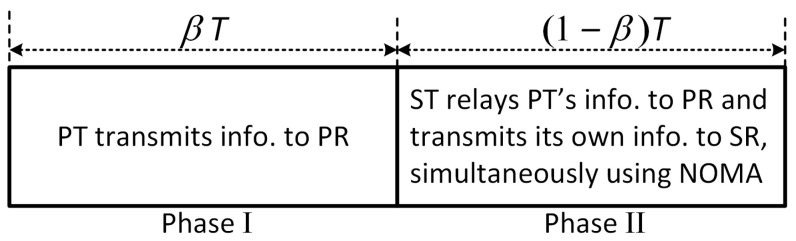
Time block of cooperative NOMA.

We consider a cooperative communication scenario as shown in Figure 1, where a primary transmitter (PT) and receiver (PR) pair is scheduled using time-division multiple access (TDMA) and a secondary transmitter (ST) is admitted to this primary link’s time slot for a certain time fraction using NOMA. For a time slot of duration *T*, the PT transmits to PR for a duration βT,0<β<1 in *phase I*, and the rest of the time-slot (1−β)T is used by ST for relaying PT’s information to the PR as well as for delivering its own information to the secondary receiver (SR) using NOMA in *phase II* (see Figure 2). In this setup, the two important parameters, having an impact on the reliability of the two links, are the allocation of time fraction to phase I and phase II, and how power is distributed to superimposed transmission of ST to PR and SR. In this paper, we assume binary phase shift keying (BPSK) modulated information. Furthermore, the channels are modeled by complex Gaussian distribution with variance $d_{i}^{-\alpha }$ where $d_{0}$, $d_{11}$, $d_{21}$ and $d_{22}$ are the distance from PT to ST, PT to PR, ST to PR, and ST to SR, respectively. Thus, each channel gain is defined as $g_{i}∼CN\left(0,d_{i}^{-\alpha }\right)$, where α>0 is the path loss exponent. Moreover, we assume that the noise in all paths follows the additive white Gaussian distribution $n_{i}∼CN\left(0,\sigma^{2}\right)$.

Hence, the received signal at the PR ($y_{PR}^{I}$) and ST ($y_{PS}$) in phase I is given by

$$y_{PR}^{I}=\sqrt{P_{p}}g_{11}x_{p}+n_{0},\;y_{PS}=\sqrt{P_{p}}g_{0}x_{p}+n_{0},$$

where $x_{p}$ is the signal transmitted by PT, and $P_{p}$ is its transmission power. Meanwhile, the signal-to-noise ratio (SNR) of each user is given as

(1) $$\gamma_{11}=\frac{E\{({\sqrt{P_{p}}g_{11}x_{p})}^{2}\}}{E\{\left({n_{0})}^{2}\right\}}=\frac{P_{p}{\left|g_{11}\right|}^{2}}{\sigma^{2}},$$

(2) $$\gamma_{0}=\frac{E\{({\sqrt{P_{p}}g_{0}x_{p})}^{2}\}}{E\{\left({n_{0})}^{2}\right\}}=\frac{P_{p}{\left|g_{0}\right|}^{2}}{\sigma^{2}},$$

with $E\left\{|x_{p}{|}^{2}\right\}=1$.

In phase II, the ST acts as a relay and uses the PF function to relay incoming information from PT to PR. In the relaying process, ST transmits PT’s information as well as its own information simultaneously using the NOMA strategy. In addition, ST uses the NOMA strategy to send information of PT and its own at the same time.

### 2.1. PF-NOMA

**Figure 3:**
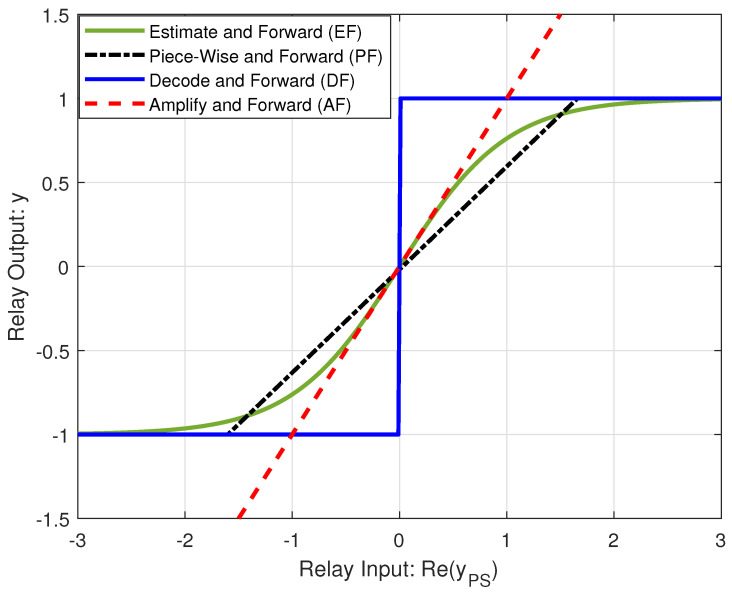
A comparison of the input–output relationship between different relaying protocols.

The optimal relay function that minimizes the error probability is shown to be the Lambert-W function in [22]. The Lambert-W function acts like DF and AF functions in high SNR and low SNR, respectively. The estimate and forward (EF) function behaves like the Lambert-W function, and it is the optimum relay function in memory-less relay networks that maximizes SNR at the destination [20]. EF has the hyperbolic tangent form and outperforms DF and AF protocol, which are two conventional relaying protocols in wireless networks. Unfortunately, due to the complicated EF function, analyzing the system performance in terms of the error probability and probability density function (PDF) is difficult. On the other hand, the authors in [21] proposed a PF function as a linear approximation of the EF by dividing the received signal into three segments. Figure 3 shows a comparison of different relaying protocols, with PF approximation outperforming the others.

As mentioned, PF contains three segments, which are defined by comparing the real value of the received signal ($R(y_{PS})$) at the ST with a threshold (Γ). If  $R(y_{PS})\leq -\Gamma$ or $R(y_{PS})\geq \Gamma$, ST acts like a DF protocol because the channel is reliable. Otherwise, ST sends the signal with an appropriate slope to the receivers based on the quality of the channel between PT and ST. Thus, the signal transmitted by ST using the PF protocol is

(3) $$\begin{array}{c} y=\left\{\begin{array}{cc} -1, & \mathrm{if}\;R(y_{PS})\leq -\Gamma \\ \frac{1}{\Gamma }\;R(y_{PS}), & \mathrm{if}\;-\Gamma <R(y_{PS})<\Gamma \\ 1, & \mathrm{if}\;R(y_{PS})\geq \Gamma , \end{array}\right. \end{array}$$

where $R\left(y_{PS}\right)=R\left(\sqrt{P_{p}}g_{0}x_{P}+n_{0}\right)=\sqrt{P_{p}}{\tilde{g}}_{0}x_{P}+{\hat{n}}_{0}\;,\;{\tilde{g}}_{0}∼N\left(0,d_{i}^{-\alpha }/2\right)$ and ${\hat{n}}_{0}∼N\left(0,\sigma^{2}/2\right)$. Since EF is the odd function, Γ can be determined by the median value of the function output in the first quadrant [21]. Thus, the value of Γ can be determined as

(4) $$\begin{array}{c} \tanh\left(\sqrt{\gamma_{0}}\frac{\Gamma }{2}\right)=\frac{1}{2}\Rightarrow \Gamma =\frac{\ln3}{\sqrt{\gamma_{0}}}. \end{array}$$

In phase II, we use PF-based relaying of the PT’s information, which is transmitted simultaneously with the ST’s information using NOMA. We term this scheme as PF-NOMA. The transmitted signal ($x_{PS}$) at the ST is given by

(5) $$\begin{array}{c} x_{PS}=\sqrt{\omega P_{S}}\frac{y}{\zeta }+\sqrt{\left(1-\omega \right)P_{S}}x_{s}, \end{array}$$

assuming that $\omega P_{s}$ is a NOMA fraction for relaying and $\left(1-\omega \right)P_{s}$ is a NOMA fraction for transmitting information of ST, $x_{s}$. Furthermore, $P_{S}$ is the transmit power at the ST and $\zeta =\sqrt{E\left\{{\left|y\right|}^{2}\right\}}$ is the normalization factor of the signal.

The received signal at the PR ($y_{PR}^{II}$) and SR ($y_{SR}$) can be written as

$$\begin{array}{c} y_{PR}^{II}=g_{21}x_{PS}+{\tilde{n}}_{0},\;y_{SR}=g_{22}x_{PS}+{\tilde{n}}_{0}. \end{array}$$

The receivers decode information based on the quality of the channel. For instance, if $|g_{21}{|}^{2}<{\left|g_{22}\right|}^{2}$, PR decodes its own message by treating SR’s message as noise and SR performs successive interference cancellation (SIC). In SIC, SR decodes $x_{p}$ first, then subtracts this message from the composite received signal before decoding its own message. For PF-NOMA, $y_{PR}^{II}$ is defined as follows

(6) $$\begin{array}{c} y_{PR}^{II}=\left\{\begin{array}{cc} \frac{1}{\zeta }g_{21}\sqrt{\omega P_{S}}+g_{21}\sqrt{\left(1-\omega \right)P_{S}}x_{S}\;+\;{\tilde{n}}_{0}, & R(y_{PS})\geq \Gamma \\ \; & \\ \frac{1}{\zeta \Gamma }g_{21}\sqrt{\omega P_{S}P_{P}}{\tilde{g}}_{0}x_{P}+g_{21}\sqrt{\left(1-\omega \right)P_{S}}x_{S}+ & -\Gamma <R(y_{PS})<\Gamma \\ g_{21}\sqrt{\omega P_{S}}\frac{{\hat{n}}_{0}}{\zeta \Gamma }+{\tilde{n}}_{0},\\ \; & \\ \frac{-1}{\zeta }g_{21}\sqrt{\omega P_{S}}+g_{21}\sqrt{\left(1-\omega \right)P_{S}}x_{S}\;+\;{\tilde{n}}_{0}, & R(y_{PS})\leq -\Gamma \end{array}\right. \end{array}$$

while $y_{SR}$ is defined as

(7) $$\begin{array}{c} y_{SR}=\left\{\begin{array}{cc} \frac{1}{\zeta }g_{22}\sqrt{\omega P_{S}}+g_{22}\sqrt{\left(1-\omega \right)P_{S}}x_{S}+{\tilde{n}}_{0}, & R(y_{PS})\geq \Gamma \\ \; & \\ \frac{1}{\zeta \Gamma }g_{22}\sqrt{\omega P_{S}P_{P}}{\tilde{g}}_{0}x_{P}+g_{22}\sqrt{\omega P_{S}}\frac{{\hat{n}}_{0}}{\zeta \Gamma }+{\tilde{n}}_{0}, & -\Gamma <R(y_{PS})<\Gamma \\ \; & \\ \frac{-1}{\zeta }g_{22}\sqrt{\omega P_{S}}+\sqrt{\left(1-\omega \right)P_{S}}x_{S}+{\tilde{n}}_{0}, & R(y_{PS})\leq -\Gamma \end{array}\right. \end{array}$$

### 2.2. Deriving SNR at the Receivers for PF-NOMA

PR and SR use SIC to decode $x_{p}$ and $x_{s}$, respectively. Therefore, the SNR at PR and SR for the condition $|g_{21}{|}^{2}\geq |g_{22}{|}^{2}$ is

(8) $$\begin{array}{l} \begin{array}{cc} \gamma_{21} & =\left\{\begin{array}{cc} \frac{\omega P_{S}{\left|g_{21}\right|}^{2}}{\zeta^{2}\sigma^{2}}, & |R(y_{PS})|\geq \Gamma \\ \frac{\omega P_{S}P_{p}|g_{21}{|}^{2}{\left|{\tilde{g}}_{0}\right|}^{2}}{\zeta^{2}\Gamma^{2}\left(\omega P_{S}\frac{\sigma^{2}}{2\zeta^{2}\Gamma^{2}}{\left|g_{21}\right|}^{2}+\sigma^{2}\right)}, & |R(y_{PS})|<\Gamma \end{array}\right.\\ \; & \end{array}\\ \begin{array}{cc} \gamma_{22} & =\left\{\begin{array}{cc} \frac{\left(1-\omega \right)P_{S}|g_{22}{|}^{2}}{|g_{22}{|}^{2}\omega P_{S}\frac{1}{\zeta^{2}}+\sigma^{2}}, & |R(y_{PS})|\geq \Gamma \\ \frac{\left(1-\omega \right)P_{S}|g_{22}{|}^{2}}{\omega P_{S}|g_{22}{|}^{2}\frac{1}{\zeta^{2}\Gamma^{2}}P_{P}\left|{\tilde{g}}_{0}{|}^{2}+\omega P_{S}\right|g_{22}{|}^{2}\frac{\sigma^{2}}{{2\zeta }^{2}\Gamma^{2}}+\sigma^{2}}, & |R(y_{PS})|<\Gamma \end{array}\right. \end{array} \end{array}$$

For the condition $|g_{21}{|}^{2}<|g_{22}{|}^{2}$, we have

(9) $$\begin{array}{l} \begin{array}{cc} \gamma_{21} & =\left\{\begin{array}{cc} \frac{\omega P_{S}|g_{21}{|}^{2}}{\zeta^{2}\left(|g_{21}{|}^{2}\left(1-\omega \right)P_{S}+\sigma^{2}\right)}, & |R(y_{PS})|\geq \Gamma \\ \frac{\omega P_{S}P_{p}\left|g_{21}{|}^{2}\right|{\tilde{g}}_{0}{|}^{2}}{\zeta^{2}\Gamma^{2}\left(\left(1-\omega \right)P_{S}\left|g_{21}{|}^{2}+\omega P_{S}\frac{\sigma^{2}}{{2\zeta }^{2}\Gamma^{2}}\right|g_{21}{|}^{2}+\sigma^{2}\right)}, & |R(y_{PS})|<\Gamma \end{array}\right.\\ \; & \end{array}\\ \begin{array}{cc} \gamma_{22} & =\left\{\begin{array}{cc} \frac{\left(1-\omega \right)P_{S}|g_{22}{|}^{2}}{\sigma^{2}}, & |R(y_{PS})|\geq \Gamma \\ \frac{\left(1-\omega \right)P_{S}|g_{22}{|}^{2}}{\omega P_{S}|g_{22}{|}^{2}\frac{1}{\zeta^{2}\Gamma^{2}}\frac{\sigma^{2}}{2}+\sigma^{2}}, & |R(y_{PS})|<\Gamma \end{array}\right. \end{array} \end{array}$$

Since PF has three segments, we have different values for SNR based on the signal that is transmitted by ST in (8) and (9). The value of threshold has an important role on the SNR, and it depends on the quality of the channel between PT and ST.

### 2.3. Rate Regions for PF-NOMA Protocol

We use PF relaying protocol at the ST node to improve the performance of the system. As mentioned in the previous section, PF acts like AF and DF in low SNR and high SNR, respectively. Thus, based on the quality of the channel between PT and ST, we construct the PF signal and superimpose it with the message of ST. At the PR, we apply maximum ratio combining (MRC), and we assume that ST uses partial repetition coding [23]. In a partial repetition coding scheme, we assume that the PT uses a fraction β of the channel and the ST (relay) uses fraction (1−β) of the channel. In a case that β>0.5, we have (1−β)<β. Thus, the relay cannot transmit all the regenerated information during the (1−β)T period. Therefore, the ST omits some part of the information and sends a fraction (1−β)/β. Vice versa, when β<0.5. When β=0.5, the ST has full cooperation like a conventional repetition scheme [23]. Thus, the rate at the PR and SR for the PF-NOMA protocol by using partial repetition coding at the relay node is [6,23]

(10) $$\begin{array}{cc} R_{P} & =\left\{\begin{array}{c} \beta \log_{2}\left(1+\gamma_{11}+\gamma_{21}\right)+\left(1-2\beta \right)\log_{2}\left(1+\gamma_{21}\right),\;\;\;\;\;\beta \in \left(0,0.5\right]\\ \left(1-\beta \right)\log_{2}\left(1+\gamma_{11}+\gamma_{21}\right)+\left(2\beta -1\right)\log_{2}\left(1+\gamma_{11}\right),\;\;\;\;\beta \in \left[0.5,1\right) \end{array}\right. \end{array}$$

(11) $$\begin{array}{cc} R_{S} & =\left(1-\beta \right)\log_{2}\left(1+\gamma_{22}\right) \end{array}$$

**Figure 4:**
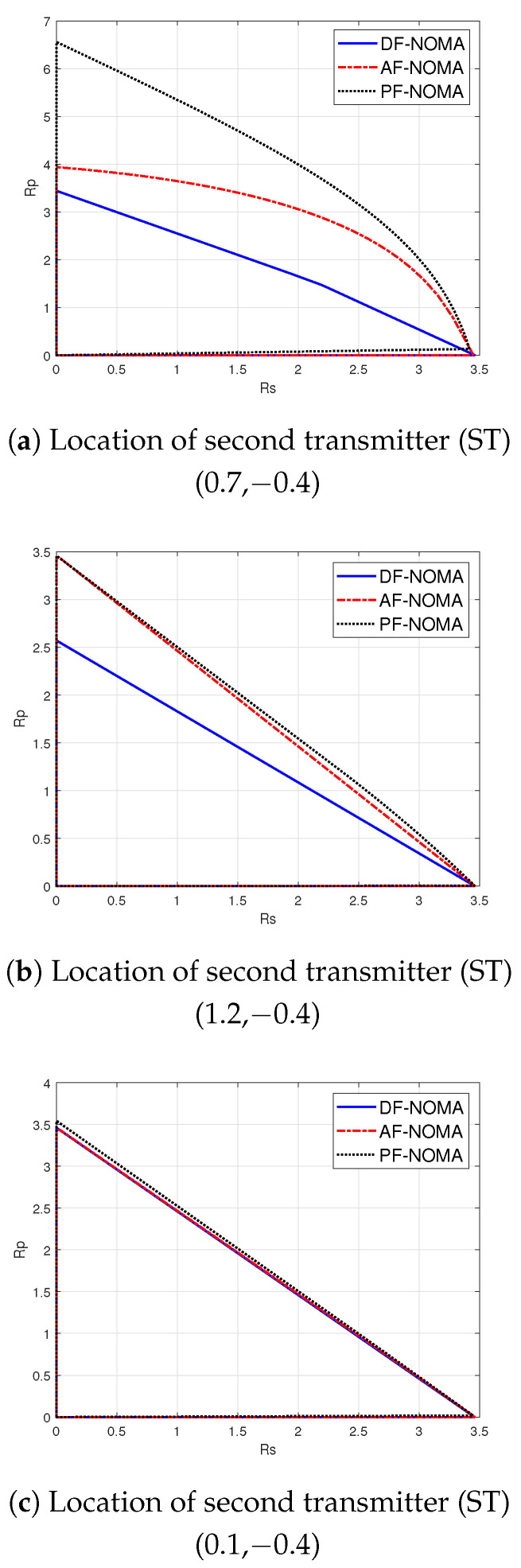
Achievable rate at the secondary receiver ($R_{s}$) versus achievable rate at the primary receiver ($R_{p}$) for PF-NOMA, AF-NOMA, and DF-NOMA schemes at different locations of the secondary transmitter.

Using (10) and (11), we plot the rate region of PF-NOMA for different ST locations in Figure 4. The figure also shows the rate regions for DF-NOMA and AF-NOMA schemes based on [6]. Figure 4 shows that the rate region for PF-NOMA remains higher than DF-NOMA and AF-NOMA schemes, while PF-NOMA significantly outperforms other schemes at certain ST locations. The simulation parameters used to obtain these results are given in Section 4, while the results are analyzed further in Section 4.1.

Motivated by these gains in using PF-NOMA, in what follows, we formulate an optimization problem to find the optimal values of β and ω that maximize the rate at SR.

## 3. Maximizing Rate at the Secondary Receiver

In this section, we define an optimization problem to maximize the rate at the secondary receiver while satisfying the rate constraints for both the primary and the secondary systems. Since the rate at the secondary receiver is a function of time fraction, β∈(0,1)={β∈R|0<β<1}, and power fraction, ω∈(0,1)={ω∈R|0<ω<1}, the optimization problem can be formulated as

(12a) $$\begin{array}{cccc} & \max_{\beta ,\omega } & \; & R_{s}\left(\beta ,\omega \right) \end{array}$$

(12b) $$\begin{array}{cccc} & s.t. & & R_{s}\geq R_{0}, \end{array}$$

(12c) $$\begin{array}{cccc} & & & R_{p}\geq R_{0}, \end{array}$$

(12d) $$\begin{array}{cccc} & & & R_{p}\geq R_{th}, \end{array}$$

(12e) $$\begin{array}{cccc} & & & 0<\beta <1, \end{array}$$

(12f) $$\begin{array}{cccc} & & & 0<\omega <1. \end{array}$$

where constraints (12b) and (12c) ensure that data rates $R_{s}$ and $R_{p}$ at both the primary and secondary receivers, respectively, satisfy a target rate $R_{0}$. Moreover, (12d) ensures that the secondary system must not degrade the performance of the primary system, where $R_{th}=\log_{2}\left(1+\gamma_{11}\right)$ with $\gamma_{11}$ the SNR of the PT-PR link. By substituting (8) and (9) in (11), we find that $R_{s}\left(\beta ,\omega \right)$ is neither a convex nor a concave function of β and ω. Thus, to solve the optimization problem in (12a), we decompose it into two sub-optimization problems as

(13a) $$\begin{array}{cc} (\mathrm{OP}–1): & \\ & \max_{\beta }\;R_{s}\left(\beta \right) \end{array}$$

(13b) $$\begin{array}{cccc} & s.t. & & R_{s}\geq R_{0}, \end{array}$$

(13c) $$\begin{array}{cccc} & & & R_{p}\;\geq \;R_{m}, \end{array}$$

(13d) $$\begin{array}{cccc} & & & 0\;<\;\beta \;<\;1, \end{array}$$

(14a) $$\begin{array}{cc} (\mathrm{OP}-2): & \\ & \max_{\omega }\;R_{s}\left(\omega \right) \end{array}$$

(14b) $$\begin{array}{cccc} & s.t. & & R_{s}\geq R_{0}, \end{array}$$

(14c) $$\begin{array}{cccc} & & & R_{p}\;\geq \;R_{m}, \end{array}$$

(14d) $$\begin{array}{cccc} & & & 0\;<\;\omega \;<\;1, \end{array}$$

where $R_{m}=\max\left\{R_{0},R_{th}\right\}$. To substantiate this decomposition, assume $\left(\beta^{*},\omega^{*}\right)$ are the joint optimum values for time and power fractions in (12a) that maximize the rate at the secondary receiver, $R_{s}\left(\beta^{*},\omega^{*}\right)$. Meanwhile, from OP-1, we can find the optimum feasible regions for time fraction $\beta^{*}$. If we assume the feasible power fraction from OP-1 is equal to another feasible ω (i.e., $\omega \neq \omega^{*}$), the maximum achievable rate is equal to $R_{s}\left(\beta^{*},\omega \right)$. Therefore, $R_{s}\left(\beta^{*},\omega \right)$ will be larger than any other value for ω, leading to $R_{s}\left(\beta^{*},\omega \right)>R_{s}\left(\beta^{*},\omega^{*}\right)$. Similarly, $R_{s}\left(\beta ,\omega^{*}\right)>R_{s}\left(\beta^{*},\omega^{*}\right)$ holds for OP-2. However, it is a contradiction because we assume that $(\beta^{*}$, $\omega^{*})$ are the optimum values of the time and power fraction in problem (12a). Thus, only joint optimal $\beta^{*}$ in OP-1 leads to $\omega^{*}$ and $\omega^{*}$ in OP-2 leads to $\beta^{*}$.

In the following subsections, we solve the problems (OP-1) and (OP-2), assuming $|g_{21}{|}^{2}\geq |g_{22}{|}^{2}$.

### 3.1. Solving OP-1

To solve OP-1, we first determine the behavior of $R_{s}$ and $R_{p}$ with respect to β from (10) and (11), respectively. For $R_{s}$ in (11), it is observed that $R_{S}$ is monotonically decreasing with β, since the function $\log_{2}\left(\cdot \right)$ is independent of β and always positive for its argument always being greater than one. On the other hand, the behavior of $R_{p}$ in (10) can be determined from its first derivative. Assuming $v_{1}=\log_{2}\left(1+\gamma_{11}+\gamma_{21}\right)$, $v_{2}=\log_{2}\left(1+\gamma_{21}\right)$, $v_{3}=\log_{2}\left(1+\gamma_{11}\right)\;$, and taking the derivative, we have

(15) $$\begin{array}{cc} \frac{\partial R_{p}}{\partial \beta } & =\left\{\begin{array}{cc} \;\;\;\;v_{1}-2v_{2}, & \beta \in (0,0.5]\\ -v_{1}+2v_{3}, & \beta \in [0.5,1). \end{array}\right. \end{array}$$

Equation (15) leads to following four monotonic conditions

(16) $$\begin{array}{cc} C_{i} & :=\left\{\begin{array}{cc} C_{1}:\;\;\;\;v_{1}-2v_{2}>0, & \mathrm{increasing}\\ C_{2}:\;\;\;\;v_{1}-2v_{2}<0, & \mathrm{decreasing}\\ C_{3}:-v_{1}+2v_{3}>0, & \mathrm{increasing}\\ C_{4}:-v_{1}+2v_{3}<0, & \mathrm{decreasing}. \end{array}\right. \end{array}$$

The solution of OP-1 lies at the intersection of four regions in (16) (i.e., $C_{1},C_{2},C_{3},C_{4}$), which requires finding the valid boundary of β for each region. The boundary values under each constraint for each region can be derived as in Table 1, which leads to the following final boundaries, respectively

(17) $$\begin{array}{c} \max\left(0,\frac{R_{m}-v_{2}}{v_{1}-2v_{2}}\right)\leq \beta \leq \min\left(\frac{1}{2},1-\frac{R_{0}}{\log_{2}\left(1+\gamma_{22}\right)}\right), \end{array}$$

(18) $$\begin{array}{c} 0<\beta \leq \min\left(\frac{1}{2},1-\frac{R_{0}}{\log_{2}\left(1+\gamma_{22}\right)},\frac{R_{m}-v_{2}}{v_{1}-2v_{2}}\right), \end{array}$$

(19) $$\begin{array}{c} \max\left(\frac{1}{2},\frac{R_{m}+v_{3}-v_{1}}{2v_{3}-v_{1}}\right)\leq \beta \leq 1-\frac{R_{0}}{\log_{2}\left(1+\gamma_{22}\right)}, \end{array}$$

(20) $$\begin{array}{c} \frac{1}{2}\leq \beta \leq \min\left(\frac{R_{m}+v_{3}-v_{1}}{2v_{3}-v_{1}},1-\frac{R_{0}}{\log_{2}\left(1+\gamma_{22}\right)}\right). \end{array}$$

Finally, the feasible region for optimization problem OP-1 is calculated by the intersection of feasible β for $C_{1},C_{2},C_{3},C_{4}$.

### 3.2. Solving OP-2

As a first step to solve OP-2 in (14a), we evaluate if $R_{s}$ is monotonically increasing or decreasing with ω. From (8) with (11), we see that $R_{s}$ behavior must be investigated for two conditions: $|R(y_{PS})|\geq \Gamma$ and $|R(y_{PS})|<\Gamma .$

#### 3.2.1. $|R(y_{PS})|\geq \Gamma$

We show that $R_{s}$ is monotonically decreasing in this condition in Appendix A. Therefore, the feasible region for ω under constraint (14b) is

(21) $$\omega \leq \frac{P_{s}|g_{22}{|}^{2}-A\left(R_{0},\beta \right)\sigma^{2}}{A\left(R_{0},\beta \right)\left|g_{22}{|}^{2}P_{s}\frac{1}{\zeta^{2}}+\right|g_{22}{|}^{2}P_{s}},$$

where $A\left(R_{0},\beta \right):=2^{R_{0}/\left(1-\beta \right)}-1$.

Similarly, we need to specify the increasing or decreasing behavior of $R_{p}$ for the constraint (14c) based on the second derivative of $R_{p}$ with respect to ω. As proved in Appendix B, $R_{p}$ is a concave function, and the feasible region of ω can be determined by using Jensen’s inequality. For any value of β, we have

(22) $$\begin{array}{c} R_{p}\leq \log_{2}\left(1+\beta \frac{P_{p}}{\sigma^{2}}|g_{11}{|}^{2}+\left(1-\beta \right)\frac{\omega P_{s}|g_{21}{|}^{2}}{\zeta^{2}\sigma^{2}}\right), \end{array}$$

(23) $$\omega \geq \frac{B\left(R_{m}\right)-\beta \frac{P_{p}}{\sigma^{2}}|g_{11}{|}^{2}\;}{\left(1-\beta \right)\frac{P_{s}|g_{21}{|}^{2}}{\zeta^{2}\sigma^{2}}},$$

where $B\left(R_{m}\right):=2^{R_{m}}-1$. Therefore, for $|R(y_{PS})|\geq \Gamma$, the final boundary of ω from (21), (23), and constraint (14d) is

(24) $$\begin{array}{cc} \; & \max\left(0,\frac{B\left(R_{m}\right)-\beta \frac{P_{p}}{\sigma^{2}}|g_{11}{|}^{2}\;}{\left(1-\beta \right)\frac{P_{s}|g_{21}{|}^{2}}{\zeta^{2}\sigma^{2}}}\right)\leq \omega \leq \min\left(1,\frac{P_{s}|g_{22}{|}^{2}-A\left(R_{0},\beta \right)\sigma^{2}}{A\left(R_{0},\beta \right)\left|g_{22}{|}^{2}P_{s}\frac{1}{\zeta^{2}}+\right|g_{22}{|}^{2}P_{s}}\right). \end{array}$$

#### 3.2.2. $|R(y_{ps})|<\Gamma$

We find in Appendix C that the first derivative of $R_{s}$ with respect to ω is always negative; thus, $R_{s}$ has a decreasing trend. This leads to the boundary of ω under constraint (14b) as in (25).

(25) $$\omega \leq \frac{P_{s}|g_{22}{|}^{2}-A\left(R_{0},\beta \right)\sigma^{2}}{P_{s}|g_{22}{|}^{2}\left(\frac{A(R_{0},\beta )}{\zeta^{2}\Gamma^{2}}P_{p}|{\tilde{g}}_{0}{|}^{2}-\frac{A\left(R_{0},\beta \right)\sigma^{2}}{{2\zeta }^{2}\Gamma^{2}}\;+\;1\right)},$$

where $A\left(R_{0},\beta \right):=2^{R_{0}/\left(1-\beta \right)}-1$.

To obtain the boundary of ω under constraint (14c), we find in Appendix D that the second derivative of $R_{p}$ based on ω is negative for β∈(0,1). Therefore, $R_{p}$ is a concave function of ω, and using Jensen’s inequality, we have

(26) $$R_{p}\leq \log_{2}\left(1+\beta \frac{P_{p}}{\sigma^{2}}|g_{11}{|}^{2}+\left(1-\beta \right)\frac{\omega P_{s}P_{p}\left|g_{21}{|}^{2}\right|{\tilde{g}}_{0}{|}^{2}}{\zeta^{2}\Gamma^{2}\left(\omega P_{s}\frac{\sigma^{2}}{{2\zeta }^{2}\Gamma^{2}}|g_{21}{|}^{2}+\sigma^{2}\right)}\;\right),$$

(27) $$\omega \geq \frac{\left(B\left(R_{m}\right)-\beta \frac{P_{p}}{\sigma^{2}}|g_{11}{|}^{2}\right)\zeta^{2}\Gamma^{2}\sigma^{2}}{P_{s}{\left|g_{21}\right|}^{2}\left(P_{p}{\tilde{g}}_{0}{|}^{2}\left(1-\beta \right)-\frac{\sigma^{2}}{2}\left(B\left(R_{m}\right)-\beta \frac{P_{p}}{\sigma^{2}}|g_{11}{|}^{2}\right)\right)}.$$

The final boundary of ω when $|R(y_{ps})|<\Gamma$ is given in (28) based on (25), (27), and constraint (14d).

(28) $$\begin{array}{cc} \; & \mathrm{if}\;\;\;\;\;P_{p}|{\tilde{g}}_{0}{|}^{2}\left(1-\beta \right)\geq \frac{\sigma^{2}}{2}\left(B\left(R_{m}\right)-\beta \frac{P_{p}}{\sigma^{2}}|g_{11}{|}^{2}\right),\\ \; & \max\;\left(\;0,\frac{\left(B\left(R_{m}\right)-\beta \frac{P_{p}}{\sigma^{2}}|g_{11}{|}^{2}\right)\zeta^{2}\Gamma^{2}\sigma^{2}}{P_{s}{\left|g_{21}\right|}^{2}\left(P_{p}{\tilde{g}}_{0}{|}^{2}\left(1\;-\;\beta \right)\;-\;\frac{\sigma^{2}}{2}\left(B\left(R_{m}\right)-\beta \frac{P_{p}}{\sigma^{2}}|g_{11}{|}^{2}\right)\right)}\right)\;\leq \;\omega \;\leq \;\min\left(\;1,\frac{P_{s}|g_{22}{|}^{2}-A\left(R_{0},\beta \right)\sigma^{2}}{P_{s}|g_{22}{|}^{2}\left(\frac{A(R_{0},\beta )}{\zeta^{2}\Gamma^{2}}P_{p}|{\tilde{g}}_{0}{|}^{2}\;-\;\frac{A\left(R_{0},\beta \right)\sigma^{2}}{{2\zeta }^{2}\Gamma^{2}}\;+\;1\right)}\right),\\ \; & \mathrm{if}\;\;\;\;\;P_{p}|{\tilde{g}}_{0}{|}^{2}\left(1-\beta \right)\leq \frac{\sigma^{2}}{2}\left(B\left(R_{m}\right)-\beta \frac{P_{p}}{\sigma^{2}}|g_{11}{|}^{2}\right),\\ \; & 0\;<\;\omega \;\leq \;\min\left(1,\frac{P_{s}|g_{22}{|}^{2}-A\left(R_{0},\beta \right)\sigma^{2}}{P_{s}|g_{22}{|}^{2}\left(\frac{A(R_{0},\beta )}{\zeta^{2}\Gamma^{2}}P_{p}|{\tilde{g}}_{0}{|}^{2}\;-\;\frac{A\left(R_{0},\beta \right)\sigma^{2}}{{2\zeta }^{2}\Gamma^{2}}\;+\;1\right)},\frac{\left(B\left(R_{m}\right)-\beta \frac{P_{p}}{\sigma^{2}}|g_{11}{|}^{2}\right)\zeta^{2}\Gamma^{2}\sigma^{2}}{P_{s}{\left|g_{21}\right|}^{2}\left(P_{p}{\tilde{g}}_{0}{|}^{2}\left(1-\beta \right)-\frac{\sigma^{2}}{2}\left(B\left(R_{m}\right)-\beta \frac{P_{p}}{\sigma^{2}}|g_{11}{|}^{2}\right)\right)}\right). \end{array}$$

### 3.3. Optimal Rate from OP-1 and OP-2

From (17)–(20), we can find the optimum $\beta^{*}$. Moreover, based on (25) and (28), we can find the optimum $\omega^{*}$. We can find the maximum rate at the second receiver by calculating $\max\left(R_{s}\left(\beta^{*}\right),R_{s}\left(\omega^{*}\right)\right)$ [6]. The Algorithm 1 for finding the maximum rate at the second receiver for each specific location is:
**Algorithm 1.** Optimization Algorithm for PF-NOMA
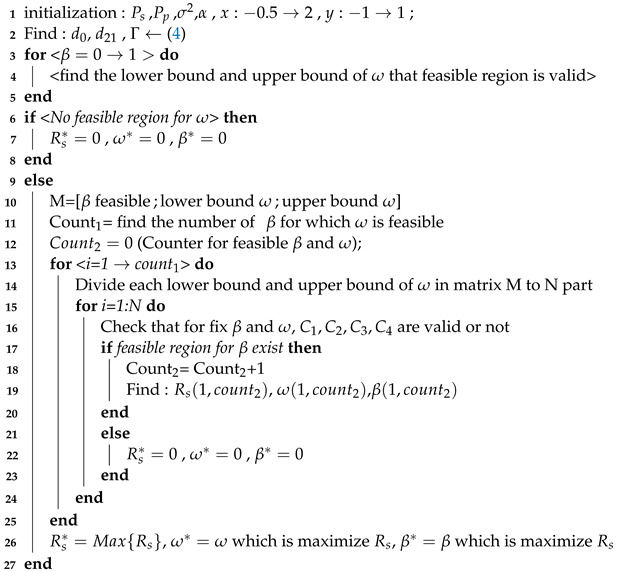



## 4. Analytical Results

In this section, we evaluate the performance of the proposed PF-NOMA scheme using MATLAB and compare it with the performance of DF-NOMA and AF-NOMA schemes studied in [6]. The simulation parameters are set as follows: $P_{p}/\sigma^{2}=10$ dB, $P_{s}/\sigma^{2}=10$ dB, α=3, ${\left|g_{i}\right|}^{2}=d_{i}^{-\alpha }$, T=1, $R_{0}=0.15\;R_{th}$, with $R_{th}=\log_{2}\left(1+\gamma_{11}\right)$. Moreover, we fix the location of PT and PR at the coordinates (0,0) and (1,0), respectively, and the distance between PT-PR and ST-SR pairs is fixed at 1 (i.e., unit-distance). In this setup, we analyze the performance of the multiple access-enabled relaying schemes by changing the relative location of the ST-SR pair with respect to the PT-PR link.

### 4.1. Rate Regions

We compare the rate regions for the proposed PF-NOMA scheme with the reference AF-NOMA and DF-NOMA schemes at three different locations of ST in Figure 4. These results are obtained by varying the values of time-fraction (β) and NOMA-fraction (ω) in (10) and (11) and taking the convex-hull of all the corresponding rates. In general, Figure 4 shows that relay location (or the link quality between PT and ST) has a critical role in the rate performance of the system. Furthermore, the PF-NOMA significantly outperforms the reference schemes for some locations. The detailed analysis of location-dependent PF-NOMA gains under optimized values of β and ω is the subject of the next section. Figure 4a shows that when the ST is located at (0.7,−0.4), PF-NOMA works better than AF-NOMA and DF-NOMA; for instance, with $R_{s}$ at 0 bit/s/Hz, PF-NOMA can yield a rate $R_{p}$ of around 6.4 bit/s/Hz, while it is around 4 and 3.45 bit/s/Hz for AF-NOMA and DF-NOMA, respectively. At this location, the PF relaying function exploits the unfavorable channel quality between PT and ST better in the decoding procedure (with higher relaying contribution of the primary transmission) than that of AF and DF functions. On the other hand, Figure 4b depicts that, for ST located at (1.2,−0.4), the further increase in the distance between PT and ST equally lowers the contributions of all relaying functions as compared to Figure 4a. Still, the rate regions for PF-NOMA and AF-NOMA, although approximately identical, remain higher compared to DF-NOMA for its performance degradation at low SNRs. Figure 4c shows that the rate regions for all the studied protocols coincide when the ST is located at (0.1,−0.4). As $d_{21}≃1=d_{11}$ in this case, the direct link between PT and PR is almost the same as the relaying link and the contributions of the relaying protocols to the primary transmission reduces.

Based on the results in Figure 4, it can be observed that when the indirect link between PT and PR is better than the direct link, an appropriate selection of the relay protocol can have a promising effect on the performance of the system.

### 4.2. Secondary Rate Maximization

**Figure 5:**
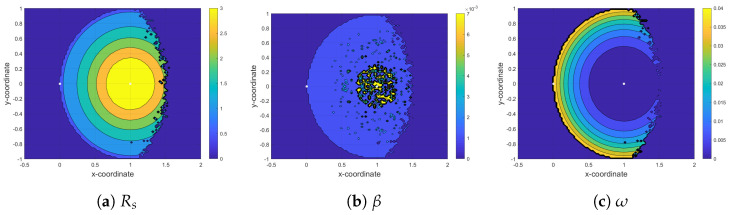
Maximizing rate at the second receiver (SR) by finding the optimum value for time fraction (β) and power fraction (ω) in the PF-NOMA protocol.

**Figure 6:**
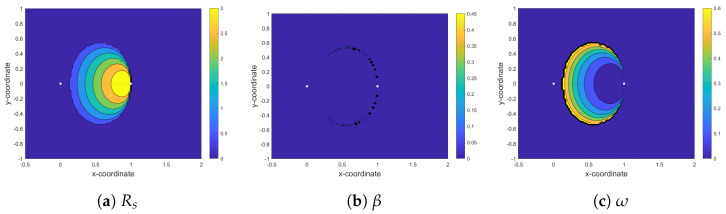
Maximizing rate at the second receiver (SR) by finding the optimum value for time fraction (β) and power fraction (ω) in the AF-NOMA protocol.

**Figure 7:**
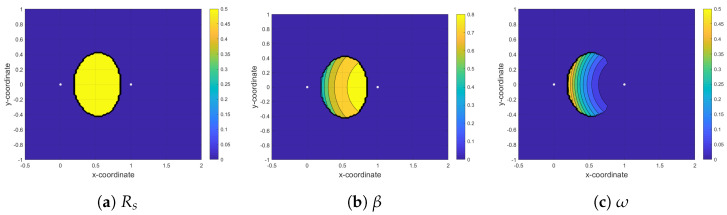
Maximizing rate at the second receiver (SR) by finding the optimum value for time fraction (β) and power fraction (ω) in the DF-NOMA protocol.

In this section, we analyze the maximum achievable rate ($R_{s}^{*}$) at the secondary receiver based on Algorithm 1, which solves the optimization defined in (12a) to obtain optimal values of time-fraction ($\beta^{*}$) in phase I and power-fraction ($\omega^{*}$) in phase II for a given ST location. Figure 5, Figure 6 and Figure 7 show the contour plots of maximum achievable rate and the corresponding optimal β and ω at the secondary receiver for PF-NOMA, AF-NOMA, and DF-NOMA schemes, respectively, with the ST location varying in x−coordinate∈[−0.5,2] and y−coordinate∈[−1,1]. Moreover, these results are obtained for a target rate at the primary and secondary receivers as $R_{0}=0.15\;R_{th}$. The color-bars in the figures show how the achievable rate and the selection of β and ω at the SR changes with the change in the ST location.

For the proposed PF-NOMA, $R_{s}^{*}$, $\beta^{*}$, and $\omega^{*}$ are plotted in Figure 5a–c, respectively. The results in Figure 5a show that whenever ST is close to PR and far from PT, despite the poor channel quality, PF-NOMA can yield a maximum rate at the secondary receiver, which is approximately 3 bits/s/Hz. This is because the PF-NOMA acts similar to AF-NOMA in low SNR regions. On the other hand, Figure 5b,c show interesting interplay between the optimal selection of the parameters $\beta^{*}$ and $\omega^{*}$ to achieve maximum rate. When ST is closer to PR, more power is assigned to $x_{s}$ than to $x_{p}$ based on (5). Since the optimization problem in (12a) is solved assuming $|g_{21}{|}^{2}>{\left|g_{22}\right|}^{2}$ (c.f., Section 3), PR always performs SIC to decode its signal $x_{p}$ from the composite signal and SR decodes $x_{s}$ directly. Meanwhile, by assigning a higher power to $x_{s}$ than $x_{p}$ at ST, the SNR at SR ($\gamma_{22}$) increases. Therefore, based on (), the rate at SR achieves a maximum value at a minimum value of ω. On the other hand, the optimum value of β in Figure 5b varies between $1\times 10^{-3}$ and $7\times 10^{-3}$. Since $R_{s}$ is monotonically decreasing with β in (11), $R_{s}$ is maximum when β is at its minimum value.

Figure 6a–c depicts the parameters of interest for AF-NOMA. As can be seen in Figure 6a, the rate at the secondary receiver increases as ST moves closer to the PR. This is because whenever ST is far from PT, the SNR of the signal at ST is low and the AF relay protocol performs better in low SNR regions. As in the case of PF-NOMA, ω and $R_{s}$ have exactly the opposite behavior. Regarding the optimum value of β in Figure 6b, the 99% of its values are less than $1\times 10^{-3}$. Since $R_{s}$ in (11) is monotonically decreasing with β, the minimum values of β lead to the maximum values of $R_{s}$. On the other hand, when β<0.5 and ST is close to PR, the condition $v_{1}-2v_{2}<0$ in ([6], Equation (16b)) holds and $R_{p}$ has a monotonically decreasing relationship with β. Hence, the value of $R_{p}$ will be high most of the time and the constraints (13c) and (14c) will be mostly satisfied. Thus, the constraint in (12a) cannot limit the maximum value of $R_{s}$.

The DF-NOMA results in Figure 7 show that when the ST is located between PT and PR, SR achieves the maximum rate and the rate has a symmetric behavior around the mid-point of the PT-PR link. In the yellow region in Figure 7a, DF can decode information with high reliability. However, Figure 7b shows that, at most of the ST locations, the optimum values of β are greater than 0.5. In DF-NOMA, we have an extra constraint in the optimization problem compared to AF-NOMA and PF-NOMA, i.e., $R_{ps}=\beta log_{2}\left(1+\gamma_{0}\right)>R_{m}$, where $R_{ps}$ implies the maximum rate at which the ST can reliably decode the primary message. Since $R_{ps}$ increases monotonically with β, β should be big enough to satisfy this condition, especially when ST is far from PT. On the other hand, $R_{s}$ has a decreasing behavior with respect to β, it asks for a trade-off between maximizing $R_{s}$ and satisfying the constraint on $R_{ps}$. Moreover, regarding Figure 7c, as long as ST becomes closer to the PR. The power that we allocate to $x_{s}$ will be increased.

**Figure 8:**
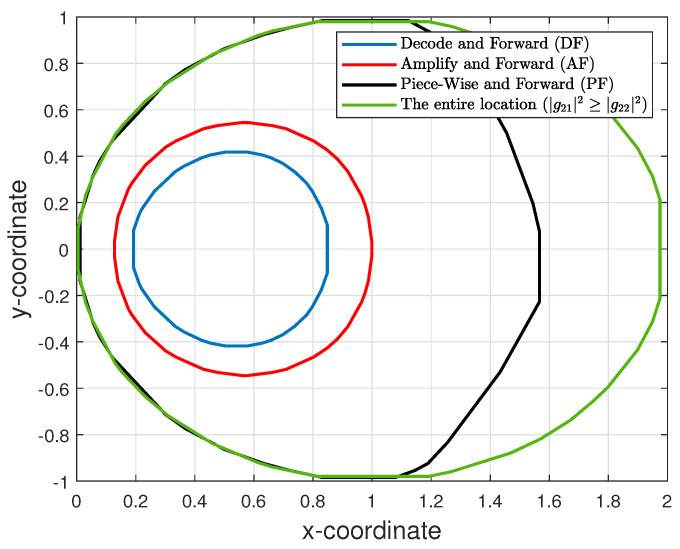
A comparison of the locations that PF-NOMA, DF-NOMA and AF-NOMA can cover for the rate greater than or equal to 0.5 bit/s/Hz at the second receiver (SR).

From these results, it can be observed that the PF-NOMA performs significantly better than other schemes in terms of degree-of-freedom in selecting relay locations to support the PT-PR link while maximizing the rate at the secondary receiver. To study this effect further, in Figure 8, we show the convex-hull of the locations that PF-NOMA, AF-NOMA and DF-NOMA can cover for the condition that the maximum rate at the secondary receiver is greater than or equal to 0.5 bit/s/Hz. In the figure, we also plot the convex-hull of the locations that satisfy $|g_{21}{|}^{2}>{\left|g_{22}\right|}^{2}$, which we define as the universal set. It can be observed that PF-NOMA can cover more locations compared to DF-NOMA and AF-NOMA, and it can almost cover most of the locations in the universal set.

### 4.3. Implementation Considerations of PF-NOMA

Like any cooperative CR-NOMA system, the proposed PF-NOMA’s implementation and operation will require overcoming many NOMA and cooperative NOMA-related challenges. Specifically, NOMA-related practical issues include (but are not limited to) decoding complexity, error propagation, power allocation complexity, and signaling and processing overhead [24]. Meanwhile, the main implementation challenges for cooperative NOMA are extra signaling procedures and overhead for coordination between the coexisting systems [25]. However, different from other CR-NOMA schemes, realizing PF relaying protocol will experience challenges similar to EF relaying protocol (c.f., PF is an approximation of EF function). Based on [26], the practical EF can be divided into two categories. The first category implements EF relaying protocol by using Wyner-Ziv code at the relay [27,28,29,30], while the second category consists of works that consider the issues related to relaying quantization as a separate problem [26,31,32]. In summary, to achieve theoretical gains depicted by the proposed method, further research is needed to address the challenges in these three above-mentioned areas.

## 5. Conclusions

In this paper, we propose PF-NOMA as a cooperative relaying scheme with joint OMA and NOMA. In this case, the primary system and secondary system share the same frequency channel by dividing the total transmission time into the two time-slots, while the secondary transmitter transmits a combination of its own message and relaying message at the same time to the receivers by using the NOMA scheme. We utilized PF relaying protocol, which has the best performance compared to two conventional relay protocols (i.e., DF and AF). We extracted the achievable rate region in PF-NOMA, and the simulation results showed the superiority of the PF-NOMA protocol compare to DF-NOMA and AF-NOMA. Then, we formulated an optimization problem to maximize the rate at the secondary receiver while guaranteeing that the rate constraints at the secondary and primary receivers are satisfied. We obtained the optimum value of time splitting and power splitting factors in the optimization problem. The results showed the impact of ST’s location on the maximum achievable rate, which is significantly higher than the other relaying protocols. Moreover, we demonstrated that PF-NOMA can support more locations of ST that can meet the maximum rate compared to two conventional relaying protocols. As future work, the performance of PF relaying-enabled cooperative CR-NOMA systems can be investigated for partial DF relaying protocols.

## Figures and Tables

**Table 1:** Boundary values of β for different conditions in OP-1.

Condition	Constraint		
	$R_{s}\geq R_{0}$	$R_{p}\geq R_{m}$	0<β<1
**$C_{1}$**	$\beta \leq 1-\frac{R_{0}}{\log_{2}\left(1+\gamma_{22}\right)}$	$\beta \geq \frac{R_{m}-v_{2}}{v_{1}-2v_{2}}$	$0<\beta <\frac{1}{2}$
**$C_{2}$**	−	$\beta \leq \frac{R_{m}-v_{2}}{v_{1}-2v_{2}}$	−
**$C_{3}$**	−	$\beta \geq \frac{R_{m}+v_{3}-v_{1}}{2v_{3}-v_{1}}$	$\frac{1}{2}\leq \beta <1$
**$C_{4}$**	−	$\beta \leq \frac{R_{m}+v_{3}-v_{1}}{2v_{3}-v_{1}}$	−

## Data Availability

Not applicable.

## Appendix A

By substituting (8) into (11) for the condition $|R(y_{PS})|\geq \Gamma$, the rate at the secondary receiver is defined as

(A1) $$\begin{array}{c} R_{s}=\left(1-\beta \right)\log_{2}\left(1+\frac{\left(1-\omega \right)P_{s}|g_{22}{|}^{2}}{|g_{22}{|}^{2}\omega P_{s}\frac{1}{\zeta^{2}}+\sigma^{2}}\right). \end{array}$$

To determine $R_{s}$ behavior with respect to ω, we find the first derivative of $\log_{2}\left(.\right)$’s argument, denoted as α, with respect ω as

(A2) $$\begin{array}{cc} \; & \frac{\partial \alpha }{\partial \omega }=\frac{{-P_{s}}^{2}\left|g_{22}{|}^{4}\frac{1}{\zeta^{2}}-P_{s}\right|g_{22}{|}^{2}\sigma^{2}}{{\left(|g_{22}{|}^{2}\omega P_{s}\frac{1}{\zeta^{2}}+\sigma^{2}\right)}^{2}}\;, \end{array}$$

which is always negative; thus, $R_{s}$ is monotonically decreasing with ω.

## Appendix B

For β∈(0,0.5] and $|R(y_{PS})|\geq \Gamma$, by substituting (1) and (8) into (10), $R_{p}$ is defined as

(A3) $$\begin{array}{cc} R_{p}= & \beta \log_{2}\left(1+\frac{P_{p}}{\sigma^{2}}|g_{11}{|}^{2}+\frac{\omega P_{S}{\left|g_{21}\right|}^{2}}{\zeta^{2}\sigma^{2}}\right)+\left(1-2\beta \right)\log_{2}\left(1+\frac{\omega P_{S}{\left|g_{21}\right|}^{2}}{\zeta^{2}\sigma^{2}}\right)\;, \end{array}$$

and its second derivative is

(A4) $$\begin{array}{cc} \frac{\partial^{2}R_{p}}{\partial \omega^{2}}= & -\beta \frac{{\left(\frac{P_{s}|g_{21}{|}^{2}}{\zeta^{2}\sigma^{2}}\right)}^{2}}{{\left(1+\frac{P_{p}}{\sigma^{2}}|g_{11}{|}^{2}+\frac{\omega P_{s}|g_{21}{|}^{2}}{\zeta^{2}\sigma^{2}}\right)}^{2}}-\left(1-2\beta \right)\frac{{\left(\frac{P_{s}|g_{21}{|}^{2}}{\zeta^{2}\sigma^{2}}\right)}^{2}}{{\left(1+\frac{\omega P_{s}|g_{21}{|}^{2}}{\zeta^{2}\sigma^{2}}\right)}^{2}}<0\;, \end{array}$$

Since β is less than or equal to 0.5, (1−2β) is always positive. Therefore, the second derivative of $R_{p}$ is negative and $R_{p}$ is a concave function.

Similarly, when β∈[0.5,1) and $|R(y_{PS})|\geq \Gamma$, by substituting (1) and (8) into (10), $R_{p}$ can be written as

(A5) $$\begin{array}{cc} R_{p}= & \left(1-\beta \right)\log_{2}\left(1+\frac{P_{p}}{\sigma^{2}}|g_{11}{|}^{2}+\frac{\omega P_{S}{\left|g_{21}\right|}^{2}}{\zeta^{2}\sigma^{2}}\right)+\left(2\beta -1\right)\log_{2}\left(1+\frac{P_{p}}{\sigma^{2}}|g_{11}{|}^{2}\right)\;, \end{array}$$

having a second derivative as

(A6) $$\begin{array}{cc} \; & \frac{\partial^{2}R_{p}}{\partial \omega^{2}}=\left(1-\beta \right)\frac{-{\left(\frac{P_{s}|g_{21}{|}^{2}}{\zeta^{2}\sigma^{2}}\right)}^{2}}{{\left(1+\frac{P_{p}}{\sigma^{2}}|g_{11}{|}^{2}+\frac{\omega P_{s}|g_{21}{|}^{2}}{\zeta^{2}\sigma^{2}}\right)}^{2}}<0\;. \end{array}$$

Since (1−β) is always positive, the second derivative of $R_{p}$ is always negative. Thus, $R_{p}$ is a concave function with ω. In general, for any values of β, $R_{p}$ is a concave function of ω.

## Appendix C

If $|R(y_{PS})|\leq \Gamma$, by substituting (8) into (11), we have

(A7) $$\begin{array}{cc} \; & R_{s}=\left(1-\beta \right)\log_{2}\left(1+\frac{\left(1-\omega \right)P_{s}|g_{22}{|}^{2}}{\omega P_{s}|g_{22}{|}^{2}\frac{1}{\zeta^{2}\Gamma^{2}}P_{p}\left|{\tilde{g}}_{0}{|}^{2}+\omega P_{s}\right|g_{22}{|}^{2}\frac{1}{\zeta^{2}\Gamma^{2}}\frac{\sigma^{2}}{2}+\sigma^{2}}\right). \end{array}$$

(A8) $$\begin{array}{cc} \; & \frac{\partial \alpha }{\partial \omega }=\frac{-P_{s}\left|g_{22}{|}^{2}\left(\omega P_{s}|g_{22}{|}^{2}\frac{1}{\zeta^{2}\Gamma^{2}}P_{p}\left|{\tilde{g}}_{0}{|}^{2}+\omega P_{s}\right|g_{22}{|}^{2}\frac{1}{\zeta^{2}\Gamma^{2}}\frac{\sigma^{2}}{2}+\sigma^{2}\right)+\left(P_{s}|g_{22}{|}^{2}\frac{1}{\zeta^{2}\Gamma^{2}}P_{p}\left|{\tilde{g}}_{0}{|}^{2}+P_{s}\right|g_{22}{|}^{2}\frac{1}{\zeta^{2}\Gamma^{2}}\frac{\sigma^{2}}{2}\right)\left(1-\omega \right)P_{s}\right|g_{22}{|}^{2}}{{\left(\omega P_{s}|g_{22}{|}^{2}\frac{1}{\zeta^{2}\Gamma^{2}}P_{p}\left|{\tilde{g}}_{0}{|}^{2}+\omega P_{s}\right|g_{22}{|}^{2}\frac{1}{\zeta^{2}\Gamma^{2}}\frac{\sigma^{2}}{2}+\sigma^{2}\right)}^{2}}. \end{array}$$

Then, the first derivative of the argument of $\log_{2}\left(\cdot \right)$ in (A7), denoted as α, is derived in (A8). Since ω varies between 0 and 1, (1−ω) and ω are always greater than 0. Thus, the first derivative of α is negative and $R_{s}$ is monotonically decreasing with respect to ω.

## Appendix D

For calculating $R_{p}$, we substitute (1) and (8) into (10) when $|R(y_{PS})|\leq \Gamma$ and β∈(0,0.5]. Thus, $R_{p}$ is defined as

(A9) $$\begin{array}{cc} R_{p}= & \beta \log_{2}\left(1+\frac{P_{p}}{\sigma^{2}}|g_{11}{|}^{2}+\frac{\omega P_{s}P_{p}\left|g_{21}{|}^{2}\right|{\tilde{g}}_{0}{|}^{2}}{\zeta^{2}\Gamma^{2}\left(\omega P_{s}\frac{1}{\zeta^{2}\Gamma^{2}}|g_{21}{|}^{2}\frac{\sigma^{2}}{2}+\sigma^{2}\right)}\right)+\\ \; & \left(1-2\beta \right)\log_{2}\left(1+\frac{\omega P_{s}P_{p}\left|g_{21}{|}^{2}\right|{\tilde{g}}_{0}{|}^{2}}{\zeta^{2}\Gamma^{2}\left(\omega P_{s}\frac{1}{\zeta^{2}\Gamma^{2}}|g_{21}{|}^{2}\frac{\sigma^{2}}{2}+\sigma^{2}\right)}\right). \end{array}$$

(A10) $$\begin{array}{cc} \; & \frac{\partial^{2}R_{p}}{\partial \omega^{2}}=\left(\beta P_{s}P_{p}\left|g_{21}{|}^{2}\right|{\tilde{g}}_{0}{|}^{2}\sigma^{2}\right)\\ \; & \frac{-2P_{s}|g_{21}{|}^{2}\frac{\sigma^{2}}{2}\left(\omega P_{s}\frac{1}{\zeta^{2}\Gamma^{2}}|g_{21}{|}^{2}\frac{\sigma^{2}}{2}+\sigma^{2}\right)-2P_{s}|g_{21}{|}^{2}\frac{\sigma^{2}}{2}\left(\omega P_{s}\frac{1}{\zeta^{2}\Gamma^{2}}|g_{21}{|}^{2}\frac{\sigma^{2}}{2}+\sigma^{2}\right)-2\omega {P_{s}}^{2}P_{p}\frac{1}{\zeta^{2}\Gamma^{2}}\left|g_{21}{|}^{4}\right|{\tilde{g}}_{0}{|}^{2}\frac{\sigma^{2}}{2}}{{\left(\zeta^{2}\Gamma^{2}{\left(\omega P_{s}\frac{1}{\zeta^{2}\Gamma^{2}}|g_{21}{|}^{2}\frac{\sigma^{2}}{2}+\sigma^{2}\right)}^{2}+\zeta^{2}\Gamma^{2}{\left(\omega P_{s}\frac{1}{\zeta^{2}\Gamma^{2}}|g_{21}{|}^{2}\frac{\sigma^{2}}{2}+\sigma^{2}\right)}^{2}\frac{P_{p}}{\sigma^{2}}|g_{11}{|}^{2}+\left(\omega P_{s}\frac{1}{\zeta^{2}\Gamma^{2}}|g_{21}{|}^{2}\frac{\sigma^{2}}{2}+\sigma^{2}\right)\left(\omega P_{s}P_{p}\left|g_{21}{|}^{2}\right|{\tilde{g}}_{0}{|}^{2}\right)\right)}^{2}}\\ \; & +\left(\left(1-2\beta \right)P_{s}P_{p}\left|g_{21}{|}^{2}\right|{\tilde{g}}_{0}{|}^{2}\sigma^{2}\right)\\ \; & \frac{-2P_{s}|g_{21}{|}^{2}\frac{\sigma^{2}}{2}\left(\omega P_{s}\frac{1}{\zeta^{2}\Gamma^{2}}|g_{21}{|}^{2}\frac{\sigma^{2}}{2}+\sigma^{2}\right)-2\omega {P_{s}}^{2}P_{p}\frac{1}{\zeta^{2}\Gamma^{2}}\left|g_{21}{|}^{4}\right|{\tilde{g}}_{0}{|}^{2}\frac{\sigma^{2}}{2}}{{\left(\zeta^{2}\Gamma^{2}{\left(\omega P_{s}\frac{1}{\zeta^{2}\Gamma^{2}}|g_{21}{|}^{2}\frac{\sigma^{2}}{2}+\sigma^{2}\right)}^{2}+\left(\omega P_{s}\frac{1}{\zeta^{2}\Gamma^{2}}|g_{21}{|}^{2}\frac{\sigma^{2}}{2}+\sigma^{2}\right)\left(\omega P_{s}P_{p}\left|g_{21}{|}^{2}\right|{\tilde{g}}_{0}{|}^{2}\right)\right)}^{2}}. \end{array}$$

The second derivative of (A9) is given in (A10), which is always negative because β is between 0 and 0.5 and ω is always positive. Thus, $R_{p}$ is a concave function of ω when $|R(y_{PS})|\leq \Gamma$ and β∈(0,0.5].

Similarly, when $|R(y_{PS})|\leq \Gamma$ and β∈[0.5,1), $R_{p}$ is

(A11) $$\begin{array}{cc} R_{p}= & \left(1-\beta \right)\log_{2}\left(1+\frac{P_{p}}{\sigma^{2}}|g_{11}{|}^{2}+\frac{\omega P_{s}P_{p}\left|g_{21}{|}^{2}\right|{\tilde{g}}_{0}{|}^{2}}{\zeta^{2}\Gamma^{2}\left(\omega P_{s}\frac{1}{\zeta^{2}\Gamma^{2}}|g_{21}{|}^{2}\frac{\sigma^{2}}{2}+\sigma^{2}\right)}\right)+\\ \; & \left(2\beta -1\right)\log_{2}\left(1+\frac{P_{p}}{\sigma^{2}}|g_{11}{|}^{2}\right), \end{array}$$

with its second derivative in (A12). Since β is between 0.5 and 1, (1−β) is always positive, and we know that ω varies between 0 and 1; therefore, (A12) is always negative, and $R_{p}$ is a concave function. In general, we can say that for any value of β, $R_{p}$ is a concave function of ω.

(A12) $$\begin{array}{cc} \; & \frac{\partial^{2}R_{p}}{\partial \omega^{2}}=\left(\left(1-\beta \right)P_{s}P_{p}\left|g_{21}{|}^{2}\right|{\tilde{g}}_{0}{|}^{2}\sigma^{2}\right)\\ \; & \frac{-2P_{s}|g_{21}{|}^{2}\frac{\sigma^{2}}{2}\left(\omega P_{s}\frac{1}{\zeta^{2}\Gamma^{2}}|g_{21}{|}^{2}\frac{\sigma^{2}}{2}+\sigma^{2}\right)-2P_{s}|g_{21}{|}^{2}\frac{\sigma^{2}}{2}\left(\omega P_{s}\frac{1}{\zeta^{2}\Gamma^{2}}|g_{21}{|}^{2}\frac{\sigma^{2}}{2}+\sigma^{2}\right)-2\omega {P_{s}}^{2}P_{p}\frac{1}{\zeta^{2}\Gamma^{2}}\left|g_{21}{|}^{4}\right|{\tilde{g}}_{0}{|}^{2}\frac{\sigma^{2}}{2}}{{\left(\zeta^{2}\Gamma^{2}{\left(\omega P_{s}\frac{1}{\zeta^{2}\Gamma^{2}}|g_{21}{|}^{2}\frac{\sigma^{2}}{2}+\sigma^{2}\right)}^{2}+\zeta^{2}\Gamma^{2}{\left(\omega P_{s}\frac{1}{\zeta^{2}\Gamma^{2}}|g_{21}{|}^{2}\frac{\sigma^{2}}{2}+\sigma^{2}\right)}^{2}\frac{P_{p}}{\sigma^{2}}|g_{11}{|}^{2}+\left(\omega P_{s}\frac{1}{\zeta^{2}\Gamma^{2}}|g_{21}{|}^{2}\frac{\sigma^{2}}{2}+\sigma^{2}\right)\left(\omega P_{s}P_{p}\left|g_{21}{|}^{2}\right|{\tilde{g}}_{0}{|}^{2}\right)\right)}^{2}}. \end{array}$$
